# Behavioral and activities of daily living inventories in the diagnosis of frontotemporal lobar degeneration and Alzheimer’s disease

**DOI:** 10.1590/S1980-57642009DN20200006

**Published:** 2008

**Authors:** Valéria Santoro Bahia, Mari-Nilva Maia da Silva, Rene Viana, Jerusa Smid, Antonio Eduardo Damin, Márcia Radanovic, Ricardo Nitrini

**Affiliations:** 1 MD, PhD. Behavioral and Cognitive Neurology Unit, Department of Neurology, Hospital das Clínicas, University of São Paulo School of Medicine, São Paulo.; 2 MD, Behavioral and Cognitive Neurology Unit, Department of Neurology, Hospital das Clínicas, University of São Paulo School of Medicine, São Paulo.; 3 Neuropsychologist, Behavioral and Cognitive Neurology Unit, Department of Neurology, Hospital das Clínicas, University of São Paulo School of Medicine, São Paulo

**Keywords:** dementia, frontotemporal lobar degeneration, Alzheimer’s disease, inventories, behavior, activities of daily living

## Abstract

The differential diagnosis between frontotemporal lobar degeneration (FTLD) and Alzheimer’s disease (AD) is often challenging.

**Objectives:**

To verify the usefulness of behavioral and activities of daily living inventories in the differential diagnosis between FTLD and AD.

**Methods:**

Caregivers of 12 patients with FTLD (nine with frontotemporal dementia, two with semantic dementia and one with progressive non-fluent aphasia) and of 12 patients with probable AD were interviewed. The Brazilian version of the Frontal Behavioral Inventory (FBI) and Disability Assessment for Dementia (DAD ) were used.

**Results:**

The mean of the MMSE score was 12.4±10.7for patients with FTLD and 11.9±6.2for patients with AD (p=0.93). Mean scores on the DAD were 33.7±27.7in patients with FTLD and 55.6±29.7in patients with AD (p=0.06), while for the FBI they were 42.6±10.0for FTLD and 16.7±11.7for AD (p<0.01).

**Conclusions:**

In this study, FBI was found to be a helpful tool for the differential diagnosis between FTLD and AD. Although the DAD was not useful in differential diagnosis in our sample we believe it to be important for measuring the severity of the disease through quantitative and qualitative assessment of functional deficits of the patients.

## Abstract (pt)

O diagnóstico diferencial entre degeneração lobar fronto-temporal (DLFT) e doença de Alzheimer (DA) pode ser difícil em alguns casos.

**Objetivos:**

Verificar a utilidade de inventários comportamentais e de vida diária no diagnóstico diferencial entre DLFT e DA.

**Métodos:**

Foram entrevistados os cuidadores de 12 pacientes com DLFT (nove com demência fronto-temporal, dois com demência semântica e um com afasia progressiva não fluente) e de 12 pacientes com DA provável. As versões brasileiras dos questionáros Frontal Behavioral Inventory (FBI) e o Disability Assessment for Dementia (DAD ).

**Resultados:**

A média da pontuação do MEEM foi 12,4±10,7 para pacientes com DLFT e 11,9±6,2 para pacientes com DA (p=0.93). A média dos escores do DAD foi de 33,7±27,7 para pacientes com DLFT e 55,6±29,7 para pacientes com DA (p=0.06), enquanto que com relação ao FBI a média da pontuação foi de 42,6±10,0 para pacientes com DLFT e 16,7±11,7 para pacientes com DA (p<0.01).

**Conclusões:**

Neste estudo, o FBI apresentou uma boa acurácia no diagnóstico diferencial entre DFT e DA. Embora o DAD não tenha se mostrado útil no diagnóstico diferencial em nossa amostra, acreditamos que essa escala seja importante para analisar qualitativamente e quantitativamente os déficits funcionais dos pacientes, auxiliando na avaliação da gravidade do quadro demencial.

Frontotemporal Lobar Degeneration (FTLD) is considered the second most common form of neurodegenerative dementia after Alzheimer’s disease (AD) in presenile individuals.^1-4^ FTLD was diagnosed in 5.1% of outpatients from the Behavioral and Cognitive Neurology Unit of Hospital das Clínicas between 1991 and 20015 and in 5% of patients with presenile dementia from the Cognitive Clinic of Santa Marcelina Hospital.^6^

FTLD includes a spectrum of behavioral and cognitive disorders characterized by degeneration of the frontal and anterior temporal lobes (Neary et al., 2005).^7^ The Consensus Criteria for FTLD^7^ distinguished three variants of FTLD which reflect the predominant locus of pathology: frontotemporal dementia (FTD), semantic dementia (SD) and progressive non-fluent aphasia (PNFA).

FTD is the most common clinical presentation.^2^ This disorder is characterized by alterations in behavior, personality and executive function. The core diagnostic features include insidious onset and gradual progression of loss of insight, early decline in social interpersonal conduct and regulation of personal conduct as well as early emotional blunting.

Two other clinical subtypes of FTLD have been characterized for the most prominent symptoms of the language dysfunction. PNFA is a disorder of expressive language, including non-fluent spontaneous speech with agrammatism or phonemic paraphasias or anomia. The core diagnostic features of the SD include progressive, fluent, empty spontaneous speech; loss of word meaning, manifested by impaired naming and comprehension; semantic paraphasias and/or perceptual disorders along with agnosia for faces and objects. Behavioral changes in SD are more common than in PNFA.^8^

AD is the most common cause of dementia^7,8^ and is characterized by progressive impairment of episodic memory and other cognitive domains such as language, visuospatial perception, praxis or executive functions, generally with well preserved social skills.^11,12^ The cognitive deficits are responsible for progressive impairment of activities of daily living.^13,14^

Until recently, DLFT was considered a rare disorder indistinguishable from AD in its early clinical stages^13^ or from psychiatric disorders, and was frequently misdiagnosed even in specialist settings.^16,17^ Accurate differential diagnosis of FTD is critical, as it has implications for heritability, prognosis, therapeutics and environmental management of patients.

Behavioral assessment is useful for diagnosing FTLD in early stages particularly in FTD cases. Liscic et al.^18^ evaluated 48 FTLD patients and 27 AD patients with confirmation by autopsy. They showed that the presence of the impulsivity, disinhibition, social withdrawal and progressive non-fluent aphasia distinguished individuals with FTLD from those with AD. The performance on tests of executive dysfunction was comparable between the FTLD and AD groups. Due to this, there are studies that have advocated the use of behavioral scales in differential diagnosis between FTD and AD, and which have considered these scales better than neuropsychological tests.^19,20^

The assessment of functional ability in dementia is essential for diagnosis,^21^ staging the severity of the disease, and to guide the caregiver’s attitude^13,22^. In FTLD patients, this assessment is very important because it evaluates the executive functions under an ecological model.^23^

The goal of the present study was to investigate the use of the Brazilian version of the Frontal Behavioral Inventory (FBI) and the Disability Assessment for Dementia (DAD) in the differential diagnosis between FTLD and AD and the applicability of these inventories.

## Methods

Patients were identified through the Behavioral and Cognitive Neurology Unit of Hospital das Clínicas, in São Paulo, Brazil. There were 12 patients who fulfilled consensus criteria^5^ for FTLD (9 FTD, 2 SD, 1 APNF) and 12 patients with probable AD according to the criteria developed by the National Institute of Neurological and Communicative Disorders and Stroke and the Alzheimer’s Disease and Related Disorders Association (NINCIDS/ADRDA).^24^

Patients with other neurological or psychiatric disorders, systemic decompensated disease, motor limitations and hearing and/ or vision impairment were excluded from this study. All FTLD patients were using neuroleptics in sufficient dose to contain states of excessive agitation and all AD patients were using cholinesterase inhibitors.

The diagnosis of the patients was made by consensus between neurologists and neuropsychologists who were blinded to the FBI and DAD scores. The patients underwent a neuropsychological evaluation at the time of the diagnosis which included the Brief Cognitive Battery^25,26^ and Mattis Dementia Rating Scale.^27^ Activities of daily living were evaluated using the Functional Activities Questionnaire (FAQ).^28^ For this study, we selected only patients with mild and moderate cognitive impairment (CDR 1 and 2).

All patients underwent structural neuroimaging (CT or MRI) and functional SPECT imaging along with a battery of routine screening blood tests to exclude treatable causes of dementia.

For assessment of the activities of daily living we used the Brazilian version of the Disability Assessment for Dementia (DAD)^29,30^ which is a informant-based scale with 40 items that examines basic activities of daily living (BADLs: hygiene, dressing, continence and eating) and instrumental activities of daily living (IADLs: meal preparation, telephoning, going on an outing, finance and correspondence, medications and leisure and housework) and each activity is analyzed in terms of components of performance: initiation, planning and organization. The total score is 100 and lower scores denote greater impairment.

The FBI^31^ is a an informant-based questionnaire with 24-items, in which twelve questions assess deficit or negative behavior (apathy, spontaneity, indifference, inflexibility, concreteness, personal neglect, disorganization, inattention, loss of insight, logopenia, verbal apraxia and perseveration) and the others assess disinhibited or positive behaviors (irritability, excessive jocularity, poor judgment, inappropriateness, impulsivity, restlessness, aggression, hyperorality, hypersexuality, utilization behavior, incontinence and alien hand).

The FBI assesses change in behavior on a 4-point scale that incorporates severity and frequency (never=0, mild or occasional=1, moderate=2, and severe or very frequently=3). The total score is based on the summing of all 24 items with a maximum score of 72.

The Brazilian version of the FBI scale (FBI) (version available at present) was translated by two researchers (RV and VSB) from English to Brazilian Portuguese and then back translated from Portuguese to English by two different researchers (MR and JS). The translators were proficient in Brazilian Portuguese and in English to ensure the fidelity of the translation. The FBI is attached at the end of this article. This is the currently available adaptation of the scale.

The FBI and DAD were administered to the principal caregiver of the patients.

Statistical analyses were conducted using BioEstat 3.0 and SPSS 10.0 software. Comparisons of frequency data for the FTLD and AD groups were performed using the Mann Whitney test. Statistical significance was established at p<0.05.

## Results

Both groups were similar in duration of disease, years of schooling and MMSE scores. Complete demographic features of the patients are displayed in Table 1. Two FTLD and two AD patients were not submitted to neuropsychological assessment due to severity of the illness.

**Table 1:** Demographic features of Frontotemporal Lobar Degeneration (FTLD) and Alzheimer's disease (AD) patients.

	FTLD	AD	P
N	12	12	
Age (years)	59.7 ± 10.4	79.7 ± 5.6	<0.01
Age at onset (years)	55.4 ± 11.7	75.3 ± 6.2	<0.01
Gender	4M / 8W	4M / 8 W	1.00
Duration of illness (years)	5.3 ± 2.1	4.4 ± 2.6	0.19
Schooling (years)	9.1 ± 6.2	5.2 ± 3.5	0.19
MMSE	12.4 ± 10.7	11.9 ± 6.2	0.93

MMSE: mini mental state examination; M: men; W: women; N: number in the sample.

With respect to FBI, the score was 42.6±10.0for the FTLD patients and 16.7±11.7for the AD patients (p<0.01). Using a FBI cut-off score of 34, the sensitivity was 100% and the specificity was 83.3% and the area under the ROC curve (Receiver Operating Characteristic) was 0.96.

The score was higher in negative behaviors than positive behaviors in both FTLD patients (p<0.01) and AD patients (p=0.01). Comparisons of the behaviors in question in both groups are showed in Tables 2 and 3.

**Table 2:** Means and standard deviations of the negative behaviors in the Frontal Behavior Inventory (FBI) in Frontotemporal Lobar Degeneration (FTLD) and Alzheimer's disease (AD) patients.

	FTLD	AD	P
	2.33 (1.15)	0.83 (1.27)	0.01
Spontaneity	2.58 (0.90)	1.16 (1.46)	0.03
Indifference	2.42 (0.99)	0.14 (0.32)	<0.01
Inflexibility	2.17 (1.11)	1.00 (1.20)	0.04
Concreteness	2.00 (1.13)	1.00 (1.13)	0.06
Personal neglect	2.67 (0.49)	1.25 (1.28)	0.01
Disorganization	2.50 (0.90)	1.25 (1.36)	0.03
Inattention	2.33 (0.98)	0.83 (1.03)	<0.01
Loss of insight	2.08 (1.08)	1.50 (1.38)	0.31
Logopenia	2.17 (0.94)	0.92 (1.24)	0.02
Verbal apraxia	1.67(1.30)	0.50 (1.00)	0.05
Perseveration	2.33(1.15)	0.67 (1.23)	0.01
Total	24.75(2.89)	12.83(4.70)	<0.01

SD: standard deviation.

**Table 3:** Means and standard deviations of the positive behaviors in the Frontal Behavior Inventory (FBI) in Frontotemporal Lobar Degeneration (FTLD) and Alzheimer's disease (AD) patients.

	FTLD	AD	P
	0.91(1.24)	1.17(1.26)	0.56
Excessive jocularity	0.33(0.88)	0.33(0.65)	0.78
Poor judgment	2.58(0.67)	1.08(1.24)	<0.01
Inappropriateness	1.25(1.42)	0.33(0.49)	0.20
Impulsivity	1.92(1.16)	0.83(1.26)	0.05
Restlessness	2.08(1.31)	0.21(0.39)	<0.01
Aggression	0.75(1.21)	0.12(0.31)	0.26
Hyperorality	1.91(1.31)	0.91(1.3)	0.10
Hypersexuality	0.33(0.49)	0.08(0.19)	0.24
Utilization behavior	1.33(1.23)	0.21(0.58)	0.01
Incontinence	1.33(1.37)	0.67(0.98)	0.27
Alien hand	0.58(1.16)	0.08(0.19)	0.43
Total	13.67(7.91)	6.75(5.66)	0.03

SD: standard deviation.

The scores on the DAD were 33.7±27.7for the FTLD patients and 55.6±29.7for the AD patients (p=0.06) (Table 4). There was no significant difference between BADLs and IADLs in individuals with FTLD (p=0.11) or individuals with AD (p=0.06).

**Table 4:** Scores and standard deviations of the Disability Assessment for Dementia (DAD ) in Frontotemporal Lobar Degeneration (FTLD) and Alzheimer's disease (AD) patients.

	FTLD (SD)	AD (SD)	P
BADLs	42.7 (26.3)	66.3 (33.5)	0.07
IADLs	26.1 (29.7)	37.6 (30.2)	0.19
Total	33.7 (27.7)	55.6 (29.7)	0.06

SD: standard deviation BADLs: Basic Activities of Daily Living; IADLs: Instrumental Activities of Daily Living.

## Discussion

In this study, the FBI efficiently distinguished FTLD from AD patients showing striking behavioral differences between the two groups. The area under the ROC curve was 0.96. The sensitivity was 100% and specificity was 83.3% for a cut-off score of 34.

There was a predominance of negative behaviors in both groups. In the FTLD group this finding may have been caused by the use of neuroleptics. Among negative behaviors, concreteness and loss of insight were equally prevalent in both FTLD and AD patients. In the positive behaviors, differences were seen only in poor judgment, impulsivity, restlessness and utilization behaviors with higher scores in FTLD.

There are various instruments available to assess behavioral symptoms in patients with dementia, the Neuropsychiatry Inventory (NPI)^32^ being one such instrument. The NPI is one the most-used questionnaires for rating behavioral and psychotic symptoms in dementia, but was not designed to elucidate the symptoms of FTLD where several major behaviors for diagnosis of the FTLD are placed within subitems and are not scored separately.

The FBI was specifically devised to assess the behavioral disturbance in FTLD.^31^ Blair et al.^33^ demonstrated that the FBI was better than the NPI at discriminating FTD patients from AD patients.

Kertesz et al.^34^ administered the FBI in patients with FTD, PNFA, AD, vascular dementia and depressive disorder. It was demonstrated that this scale correctly classified 92.7% of the patients with FTD with a high internal consistency (Cronbach alpha of 0.89) and inter-rater reliability (Cohen’s Kappa of 0.90). These results were confirmed in a study on the validity of the Italian version of the FBI.^33^ In a previous Brazilian study, Caixeta^36^ used a preliminary version of the FBI test and found it to be reliable in the differential diagnosis of FTLD and AD.

The DAD was also not designed to diagnose or distinguish between different types of dementia. However, as this scale assesses activities that are related to the executive functions it was reasonable to suppose that the DAD could be used in the differential diagnosis of AD and FTLD. In our study, although DAD scores did not distinguish FTLD in AD patients, FTLD patients tended to present worse performance in activities of daily living compared to AD patients.

We believe the DAD to be important to measure the severity of the disease through quantitative and qualitative assessment of functional dysfunction impairment of the patients and not necessarily to differentiate between different types of dementia.

The DAD scale has recently undergone transcultural adaptation for Brazil, where after a back translation process, the DAD was applied to caregivers of 29 AD patients, yielding high rates of correlation and inter and intra-examiner reliability.^30^

Mioshi et al.^23^ used the DAD to quantify the impact on ADLs in different forms of FTLD (FTD, SD and PNFA) compared to AD patients. They demonstrated that the FTD was the most affected group in BADLs and IADLs whereas PNFA and SD patients were less impaired while AD lay between the two. Difficulties in performing ADLs are progressive in AD and involve IADLs to a greater extent than BADLs, in the early stages.^37^

The main limitations of our study were the very small number of the patients and the lack of autopsy confirmation of the cases.

Our findings support the use of the Brazilian version of the FBI for the differential diagnosis between AD and FTLD.

## Inventário Comportamental Frontal (FBI)

**Table:**

Nome do paciente________________________________________________________________________________________________________________________________________________________________________________________				
Registro ______________________Escolaridade_____________________________				
Cuidador________________________________________________________________________________________________________________________________________________________________________________________________				
Parentesco_____________________Escolaridade_____________________________				
Data__________________________Avaliador________________________________				
Explique ao cuidador que você está procurando mudanças de comportamento e personalidade. Pergunte ao cuidador, estas questões, na ausência do paciente. Elabore-as, se necessário. Ao final de cada questão, indague sobre a extensão de mudança comportamental e pontue de acordo com o que segue: 0=nenhum; 1=leve, ocasional; 2=moderado; 3=grave, na maioria das vezes.				
	**Nenhum 0**	**Leve,ocasional 1**	**Moderado 2**	**Grave, na maioria das vezes 3**
1. **Apatia:** Ele/ ela perdeu o interesse pelos amigos ou por atividades diárias?				
2. **Espontaneidade:** Ele/ela inicia atividades por si mesmo ou tem que ser solicitado?				
3. **Indiferença, embotamento emocional:** Ele/ela são responsivos a ocasiões de alegria ou de tristeza tanto quanto antes, ele/ ela perdeu a reatividade emocional?				
4.**Inflexibilidade:** Ele consegue mudar de decisão com coerência ou parece teimoso ou rígido quanto ao pensamento ultimamente?				
5. **Pensamento concreto:** Ele/ela interpreta propriadamente o que está sendo dito ou opta somente por significados concretos?				
6. **Negligência pessoal:** Ele/ela cuida da sua própria higiene pessoal e aparência como fazia antes?				
7. **Desorganização:** Ele/ela pode planejar e organizar atividades complexas ou se distrai facilmente, é não persistente ou incapaz de terminar um trabalho?				
8. **Inatenção:** Ele/ela consegue prestar atenção ao que está acontecendo ou parece que ele perde o foco ou nem consegue segui-lo?				
9. **Perda de insight:** Ele/ela é consciente de seus problemas ou mudanças ou parece não os perceber e até os nega quando são comentados?				
10. **Logopenia:** Ele/ela está tão falante quanto antes ou a quantidade da fala diminuiu significativamente?				
11. **Apraxia verbal:** Ele/ela tem falado com clareza ou tem cometido erros de fala? Há dificuldades na articulação da fala ou hesitação?				
12. **Perseveração:** Ele/ela repete ou persevera ações ou comentários?				
13. **Irritabilidade:** Ele/ela tem estado irritado(a) ou de “pavio-curto” ou está reagindo ao estresse ou frustração como sempre fez?				
14. **Jocosidade excessiva:** Ele/ela faz piadas ofensivas ou em excesso ou na hora errada?				
15. **Pobreza de julgamento:** Ele/ela tem tido um bom julgamento em decisões ou ações ou tem agido com falta de responsabilidade, negligência ou pobreza de julgamento?				
16. **Inadequação:** Ele/ela tem respeitado regras sociais ou tem dito ou feito coisas inaceitáveis? Ele/ ela tem sido rude ou pueril?				
17. **Impulsividade:** Ele/ela tem agido ou falado sem pensar nas conseqüências, no impulso do momento ?				
18. **Agitação:** Ele/ela tem estado agitado ou hiperativo ou seu nível de atividade está normal?				
19. **Agressividade:** Ele/ela tem mostrado agressividade ou gritado com alguém ou machucado alguém?				
20. **Hiperoralidade:** Ele/ela está bebendo mais do que o usual, comendo em excesso qualquer coisa que veja ou então colocando objetos em sua boca?				
21. **Hipersexualidade:** O comportamento sexual tem estado fora do usual ou excessivo?				
22. **Comportamento de utilização:** Ele/ela tem necessidade de tocar, sentir, examinar ou pegar objetos que estão ao alcance das mãos ou da visão?				
23. **Incontinência:** Ele/ela tem urinado ou defecado na roupa (excluindo doenças físicas, tais como infecção urinária ou imobilidade)?				
24. **Mão alienígena:** Ele tem algum problema em usar uma mão, e isto interfere com a outra mão (excluindo artrite, trauma, paralisia, etc.)?				
